# A Systematic Review and Meta‐Analysis of Footbath Effects and Optimal Procedures to Improve Sleep in Older Adults

**DOI:** 10.1111/scs.70118

**Published:** 2025-09-19

**Authors:** Shih‐Yu Chang, Yi‐Chih Lee, Hsiao‐Ying Hung, Chia‐Te Chen, Ching‐Ju Fang, Yen‐Chin Chen

**Affiliations:** ^1^ Department of Nursing College of Medicine, National Cheng Kung University Tainan Taiwan; ^2^ Department of Nursing, National Cheng Kung University, Hospital, College of Medicine National Cheng Kung University Tainan Taiwan; ^3^ Graduate Institute of Clinical Nursing, College of Medicine National Chung Hsing University Taichung Taiwan; ^4^ Department of Secretariat, National Cheng Kung University, Hospital, College of Medicine National Cheng Kung University Tainan Taiwan; ^5^ Medical Library National Cheng Kung University Tainan Taiwan; ^6^ School of Medicine, College of Medicine National Sun Yat‐Sen University Kaohsiung Taiwan

**Keywords:** footbaths, meta‐analysis, older adults, sleep quality, systematic review

## Abstract

**Aims and Objectives:**

Sleep disturbances are prevalent among older adults and are linked to adverse health outcomes. While warm footbaths are widely practised as a non‐pharmacological intervention, their effectiveness and optimal parameters remain uncertain. This systematic review and meta‐analysis aimed to evaluate the effects of footbaths on sleep quality and determine evidence‐based procedural recommendations.

**Methodological Design and Justification:**

Nine databases were systematically searched up to November 2023. Study quality was assessed using the Joanna Briggs Institute tools, and certainty of evidence was graded via the Grading of Recommendations Assessment, Development, and Evaluation (GRADE). Meta‐analyses were conducted using random‐effects models.

**Results:**

A total of 18 studies involving 950 older adults (aged ≥ 60 years; 52.3% female) were included. Warm footbaths significantly improved subjective sleep quality compared with non‐footbath controls (SMD = −0.76; 95% CI: [−1.22, −0.29]; *p* = 0.001). Optimal effects were observed with water temperatures ≤ 40°C (SMD = −1.31; 95% CI: [−1.63, −0.99]; *p* < 0.001), immersion times ≥ 10 min (SMD = −0.96; 95% CI: [−1.36, −0.57]; *p* < 0.001) and intervention periods of at least one week (SMD = −1.06; 95% CI: [−1.64, −0.48]; *p* = 0.0003), which showed significant sleep quality improvements.

**Conclusion:**

Warm footbaths before bedtime may enhance perceived sleep quality in older adults, offering a simple, culturally adaptable intervention. Standardised protocols could strengthen their role in sleep hygiene practices. Future large‐scale, multicentre trials integrating subjective and objective assessments are warranted to confirm efficacy and explore cross‐cultural applicability.

**Trial Registration:**

International Platform of Registered Systematic Review and Meta‐analysis Protocols (INPLASY): INPLASY202290025

## 1 Introduction

Sleep disturbances are highly prevalent among older adults, with the National Sleep Foundation reporting that 30%–50% of this population experience difficulties such as initiating sleep, frequent awakenings or early morning arousals [1, 2]. These disturbances are associated with age‐related changes in circadian rhythms, reduced melatonin secretion and a higher prevalence of chronic conditions [3, 4, 5]. While pharmacological treatments, including sleep medications, are commonly prescribed, approximately 76% of older adults report limited improvement, and adverse effects are frequent—particularly in those with polypharmacy [6]. Such adverse effects include daytime sedation, dizziness, cognitive impairment, confusion and an increased risk of falls [7]. As a result, nonpharmacological interventions have garnered increasing attention as safer and more sustainable alternatives for improving sleep quality in this population [8, 9, 10].

A warm footbath, recognised as a feasible and easily implemented non‐pharmacological intervention, has been proposed to improve sleep quality among older adults and can be self‐administered with adequate instruction [11, 12, 13]. Guided by Kolcaba's comfort theory, this intervention could provide multi‐dimensional comfort across physical, psychospiritual and environmental domains [14]. Physiologically, footbaths help regulate core body temperature through distal vasodilation and promote autonomic balance, facilitating physical relaxation and preparing the body for sleep [15, 16]. Psychospiritually, footbaths foster calmness and alleviate anxiety. Environmentally, they establish a ritualised self‐care routine that supports emotional regulation and readiness for sleep.

Although prior systematic reviews have examined the effects of footbaths on sleep in older adults, the evidence remains limited and inconclusive. For instance, Nasiri et al. (2024) systematically reviewed the impact of footbaths on sleep quality but provided only a narrative synthesis without quantitative effect size estimation, leaving the magnitude of benefits unclear [13]. Jiang et al. (2023) performed a meta‐analysis focusing on foot thermal therapy parameters and reported that bedtime footbaths at 40°C for ≤ 20 min and with a 10 cm heating height improved subjective sleep quality in older adults. However, several relevant studies were not included, underscoring the need for an updated review to provide a more comprehensive synthesis of the available evidence [17]. To address this gap, a systematic review and meta‐analysis were performed to evaluate the effectiveness of footbaths on sleep quality in older adults and to offer comprehensive insights for developing evidence‐based guidelines for their optimal use.

## 2 Material and Methods

### 2.1 Study Design

This systematic review and meta‐analysis was conducted in accordance with the Preferred Reporting Items for Systematic Reviews and Meta‐Analyses (PRISMA) guidelines (Table S1) [18, 19]. The review question was defined using the PICO framework: Population (P): individuals aged 60 years and older; Intervention (*I*): footbath therapy involving immersion of the feet in warm water; Comparison (C): no footbath, including routine care or other interventions; and Outcome (O): night sleep quality assessed using subjective or objective instruments. The protocol was prospectively registered with the International Platform of Registered Systematic Review and Meta‐analysis Protocol.

### 2.2 Search Strategy

Following the PICO framework, keywords were expanded to include relevant synonyms and MeSH terms. A systematic search was then performed across nine databases—MEDLINE (via Ovid), Embase, Cochrane Library, CINAHL (via EBSCOhost), Scopus, Web of Science, Airiti Library, China National Knowledge Infrastructure and Google Scholar—using Boolean operators “OR” and “AND.” The search covered all publications up to November 2023. The search strategies used were the following: (elder* OR aged OR aging OR geriatric* OR gerontolog* OR senior* OR senium* OR “old age*”) and (footbath* OR bath* OR “hot spring*” OR “geothermal spring*” OR hydrotherap* OR spa OR spas OR balneotherap* OR “balneo therap*” OR balneolog* OR sauna* OR warm* OR heat* OR hyperthermia OR thermotherap* OR “high temperature*” OR “hot temperature*”) and (sleep* OR insomni* OR dyssomni* OR agrypni* OR hyposomni* OR sopor* OR parasomni* OR polysomnograph*) AND (“randomized controlled trial” OR “controlled clinical trial” OR “randomization” OR “RCT” OR “quasi‐experimental studies”). Furthermore, the reference lists of all retrieved articles were screened to identify additional potentially relevant studies. The complete search strategy is provided in Table S2.

### 2.3 Study Selection

**FIGURE 1:**
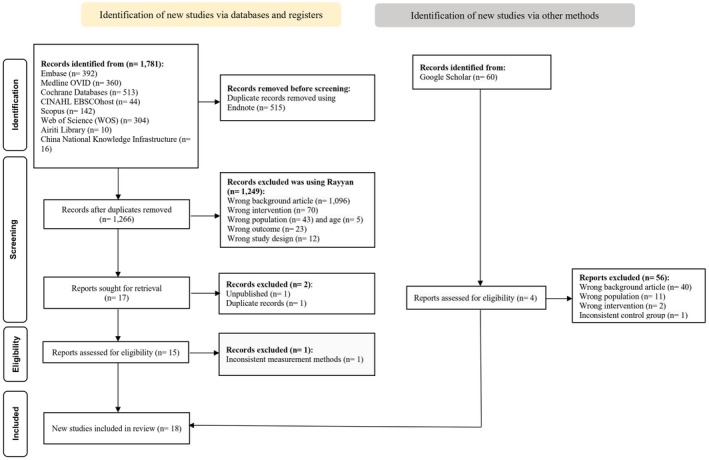
Flowchart of study selection.

After duplicate records were removed using EndNote, two reviewers independently screened the titles and abstracts, followed by full‐text review based on predefined inclusion and exclusion criteria. Studies were included if they: (1) enrolled participants aged 60 years or older; (2) evaluated footbath interventions involving immersion of the feet in warm water; (3) assessed sleep quality using objective or subjective measures; and (4) had a study design of randomised controlled trials (RCTs), randomised crossover trials or quasi‐experimental studies, with no language restrictions. Studies were excluded if full‐text articles were unavailable or if data were insufficient for analysis. Disagreements during the selection process were resolved through discussion with a third reviewer. The study selection process is illustrated in the PRISMA flowchart (Figure 1).

### 2.4 Data Extraction

Two reviewers independently extracted data using a standardised matrix table. Extracted items included author name, publication year, country, study design, sample size, participant age, female ratio, intervention details, footbath procedures, comparator and sleep outcome measures. The final dataset was verified by a third reviewer. Detailed information can be found in Table 1.

**TABLE 1:** Characteristics of the included studies.

Author (year), Country	Study design	Sample size (I1/I2/C)	Age	Female %	*I*	Footbath intervention						Control	Outcome Measure
						Water temperature	Immersive depth	Timing	Immersive time	Frequency	Duration		
Liao et al. (2008), Taiwan	Crossover	15 (15/0/0)	60–75	60	*I* ^1^: footbath	41°C	20 cm above ankle	1 h before bedtime	40 min	1time	1 day	No footbath	PSG
Huang (2009), China	RCT	60 (30/0/30)	69–80	20	*I* ^1^: footbath	40°C–45°C	Up to the ankle	30 min‐1 h before bedtime	20–30 min	Everyday	1 week	Estazolam	Total sleep time
Seo and Sohng (2011), Korea	RCT	50 (27/0/23)	71–87	90	*I* ^1^: footbath	42°C	20 cm above ankle	30 min before bedtime	30 min	Everyday	3 days	No footbath	ATG
Namba et al. (2012), Japan	Crossover	6 (6/0/0)	65 ± 5	50	*I* ^1^: footbath	40°C	Up to the ankle	NA	10 min	1 time	1 day	No footbath	PSG
Seyyedrasooli et al. (2013), Iran	RCT	46 (23/0/23)	60–75	0	*I* ^1^: footbath	41°C–42°C	10 cm depth	1 h before bedtime	20 min	Everyday	6 weeks	No footbath	PSQI
Valizadeh et al. (2015), Iran	RCT	69 (23/23/23)	60–75	0	*I* ^1^: footbath	41°C–42°C	10 cm depth	1 h before bedtime	20 min	Everyday	6 weeks	No footbath	PSQI
					*I* ^2^: foot reflexology								
Kim et al. (2016), Korea	Quasiexperimental design	30 (10/10/10)	> 65	80	*I* ^1^: footbath	40°C	20 cm above ankle	1.5 h before bedtime	30 min	Everyday	4 weeks	No footbath	ATG
					*I* ^2^: footbath	36.5°C							
Kang and Kim (2017), Korea	Quasiexperimental design	43 (20/23/0)	≥ 65	72	*I* ^1^: footbath	40°C	20 cm from ankle	1 h before bedtime	20 min	Everyday	5 days	Hand bath	KMLSEQ
Prasad and Gireesh (2018), India	Quasiexperimental design	60 (30/0/30)	60–89	83	*I* ^1^: footbath	37°C–40°C	NA	NA	10 min	Everyday	3 weeks	No footbath	PSQI
Opiña et al. (2018), Philippines	Single group Pre‐post design	16 (16/0/0)	65–86	100	*I* ^1^: footbath	40°C	NA	1 h before bedtime	20 min	1 time	2 weeks	No footbath	PSQI
Malarvizhi and Karthi (2019), India	Quasiexperimental design	60 (30/0/30)	NA	NA	*I* ^1^: footbath	NA	NA	NA	15 min	Everyday	7 days	No footbath	GSQS
Zhang (2019), China	RCT	104 (52/0/52)	60–84	46	*I* ^1^: footbath	40°C	Up to the ankle	NA	30 min	10 days per treatment course	30 days	No footbath	PSQI
Armat et al. (2021), Iran	RCT	45 (15/15/15)	≥ 60	62	*I* ^1^: footbath	40°C	10 cm above ankle	1 h before bedtime	10 min	Everyday	2 weeks	No footbath	PSQI
					*I* ^2^: footbath	37°C							
Patel and Baria (2021), India	Quasiexperimental design	120 (60/0/60)	≥ 60	38	*I* ^1^: footbath	35°C–40°C	NA	NA	NA	Everyday	7 days	No footbath	GSQS
Raut et al. (2021), India	Quasiexperimental design	60 (30/0/30)	61–70	NA	*I* ^1^: footbath	NA	NA	NA	15–20 min	Everyday	5 days	No footbath	GSQS
Wang et al. (2021), China	RCT	32 (16/0/16)	62–80	47	*I* ^1^: footbath	42°C	NA	NA	30 min	Everyday	4 weeks	Estazolam	PSQI
Guven (2022), Turkey	RCT	100 (50/50/0)	65–75	57	*I* ^1^: footbath	41°C–42°C	20 cm above ankle	1 h before bedtime	20 min	Everyday	6 weeks	Foot reflexology	PSQI
Surahmi (2022), Indonesia	Single group Pre‐post design	34 (34/0/0)	60–74	32	*I* ^1^: footbath	NA	NA	NA	20 min	Everyday	1 week	No footbath	PSQI

Abbreviations: ATG, Actigraphy; C, control; F, Female; GSQS, Groningen Sleep Quality Scale; *I*, intervention; KMLSEQ, Korean version of the Modified Leeds Sleep Evaluation Questionnaire; NA, Not applicable; PSG, Polysomnography; PSQI, Pittsburgh Sleep Quality Index; Quasi‐RCT, Quasirandomized controlled trial; RCT, Randomised controlled trial; REM, Rapid‐eye‐movement; SD, standard deviation.

### 2.5 Quality Assessment

The methodological quality of the included studies was assessed using the Joanna Briggs Institute (JBI) Critical Appraisal Checklist for RCTs and quasi‐experimental studies [20, 21]. The checklist for RCTs comprised 13 items assessing participant selection and allocation bias (Q1–Q3), bias in intervention administration (Q4–Q6), outcome assessment and measurement bias (Q7–Q9), participant retention bias (Q10) and statistical conclusion validity (Q11–Q13).

For quasi‐experimental studies, a 9‐item JBI Critical Appraisal Checklist was applied to evaluate methodological quality, covering potential sources of bias such as selection bias (Q 1, 2, 4), performance bias (Q 3), detection bias (Q 5, 7, 8), attrition bias (Q 6) and issues related to statistical conclusion validity (Q 9). Two reviewers independently conducted the quality assessment. Disagreements were resolved in a consensus meeting with the third and fourth reviewers.

### 2.6 Reporting Bias

Reporting bias was primarily assessed by constructing funnel plots and comparing trial protocols with published results. For studies without registered protocols, consistency between the Methods and Results sections was examined [19, 20].

### 2.7 Grading of the Certainty of Evidence (CoE)

The CoE for each study outcome was rated using the Grading of Recommendations Assessment, Development, and Evaluation (GRADE) methodology. Based on the GRADE domains—risk of bias, consistency, directness, precision and publication bias—the CoE was categorised as high, moderate, low or very low [22].

### 2.8 Data Synthesis/Analysis

Meta‐analyses were performed using Review Manager (RevMan) version 5.4. Statistical heterogeneity was assessed with the chi‐square test (Cochran's *Q*) and quantified using the *I* ^2^ statistic, with *p* < 0.05 indicating significant heterogeneity. Owing to variations in footbath interventions and outcome measures across studies, a random‐effects model was applied, and results were expressed as standardised mean differences (SMDs) with 95% confidence intervals (CIs). Furthermore, subgroup analyses were conducted to explore potential sources of heterogeneity and examine whether the effectiveness of footbaths varied on the basis of differences in intervention parameters, including water temperature, immersion time, total duration and foot immersion depth [23, 24]. The subgroup criteria were primarily determined based on the most frequently reported values in the included studies, supplemented by evidence from the original literature to justify certain cutoff points. For example, Liao et al. [25] reported that a water temperature of 41°C was slightly more effective than 40°C in enhancing the distal–proximal skin temperature gradient. Additionally, a systematic review reported that footbaths lasting 10–30 min were effective in improving sleep quality in older adults [13]. The meta‐analysis results are presented as forest plots [19].

## 3 Results

### 3.1 Search Results

Figure 1 presents the PRISMA flowchart of the study selection process. A total of 1781 articles were retrieved from nine databases. After removing 515 duplicates, 1266 records were screened, and 1249 were excluded as irrelevant. Seventeen full‐text articles were assessed for eligibility; three were excluded (unpublished, *n* = 1; duplicate, *n* = 1; inconsistent measures, *n* = 1). An additional four relevant articles were identified through Google Scholar. In total, 18 experimental studies met the inclusion criteria for qualitative synthesis [8, 11, 26, 27, 28, 29, 30, 31, 32, 33, 34, 35, 36, 37, 38, 39, 40, 41].

### 3.2 Characteristics of the Included Studies

The 18 included studies comprised eight RCTs [8, 11, 26, 27, 28, 29, 30, 31], six quasi‐experimental studies [32, 33, 34, 35, 36, 37], two crossover designs [38, 39] and two single‐group pre–post studies [40, 41]. A total of 950 participants were included in the analysis. As shown in Table 1, most study participants were female (52.3%), whereas two studies from Iran exclusively enrolled males [8, 26]. Among these studies, four were conducted in India (*n* = 4); three each in China (*n* = 3), Korea (*n* = 3) and Iran (*n* = 3); and one each in Taiwan (*n* = 1), Japan (*n* = 1), the Philippines (*n* = 1), Turkey (*n* = 1) and Indonesia (*n* = 1). Sample sizes ranged from 6 to 120 participants, with participant ages between 60 and 89 years.

### 3.3 Footbath Interventions

Table 1 summarises the parameters of the footbath interventions across all 18 included studies, detailing water temperature, immersion depth, timing, duration, frequency and total intervention duration. The water temperatures ranged from 35°C to 45°C. Six studies reported an immersion depth from 10 cm above the ankle to 20 cm (*n* = 6), three reported immersion up to the ankle, and two indicated a depth of 10 cm (*n* = 3). The remaining seven studies did not specify immersion depth (*n* = 7). Most interventions (*n* = 10) were administered prior to bedtime, with nine studies implementing footbaths 30 to 60 min before sleep and one study 1.5 h before bedtime. Daily footbaths were reported in 14 studies. Of the 18 studies, one did not report immersion time; the remaining studies reported durations ranging from ≤ 20 min (*n* = 11) to 40 min (*n* = 1).

### 3.4 Control Group

Among the studies included in the analysis, 14 employed routine care without footbaths as the control intervention. The remaining studies used active comparators, including foot reflexology (*n* = 1), hand baths (*n* = 1) and Estazolam administration (*n* = 2). The inclusion of both passive controls (no intervention) and active comparators enabled a broader evaluation of footbath effectiveness relative to no treatment and to other therapeutic interventions.

### 3.5 Outcome Measures

As shown in Table 1, sleep quality was assessed using both objective and subjective methods across the included studies. Objective measures included polysomnography (PSG) (*n* = 2), actigraphy (ATG) (*n* = 2) and total sleep time recordings (*n* = 1). Subjective measures consisted of self‐reported questionnaires used in 13 studies, including the Pittsburgh Sleep Quality Index (PSQI) (*n* = 9), the Groningen Sleep Quality Scale (GSQS) (*n* = 3) and the Korean Modified Leeds Sleep Evaluation Questionnaire (KMLSEQ) (*n* = 1). Most questionnaires scored lower values to indicate higher sleep quality, except for the KMLSEQ, where higher scores indicated better sleep quality.

### 3.6 Quality of the Included Studies

As shown in Table 2, the JBI checklist was applied to assess the quality of 8 RCTs and 10 quasi‐experimental studies. For the RCTs, as shown in Table 2A, the majority of items were rated ‘Yes’, indicating a low risk of bias. However, several studies received ‘Unclear’ or ‘No’ ratings for selection, performance and detection biases, primarily due to insufficient reporting of randomisation and allocation concealment, as well as a lack of blinding of participants, intervention providers and outcome assessors. In contrast, participant retention and statistical conclusion validity were generally well addressed in these trials. For the quasi‐experimental studies, as shown in Table 2B, two studies met all JBI quality criteria [32, 36]. The others received ‘Unclear’ ratings in multiple domains due to limited reporting on participant selection, intervention delivery and outcome measurement. One study [37] was rated ‘No’ in several domains because of an overall weak research design. Nevertheless, most studies adequately addressed attrition bias and statistical conclusion validity.

**TABLE 2:** JBI checklist for randomised controlled trials (RCTs) and quasi‐experimental studies.

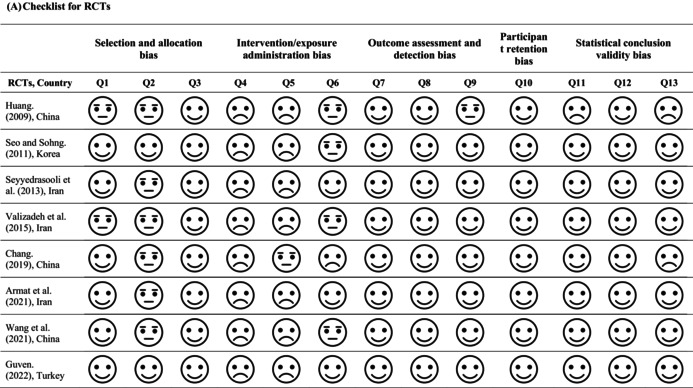

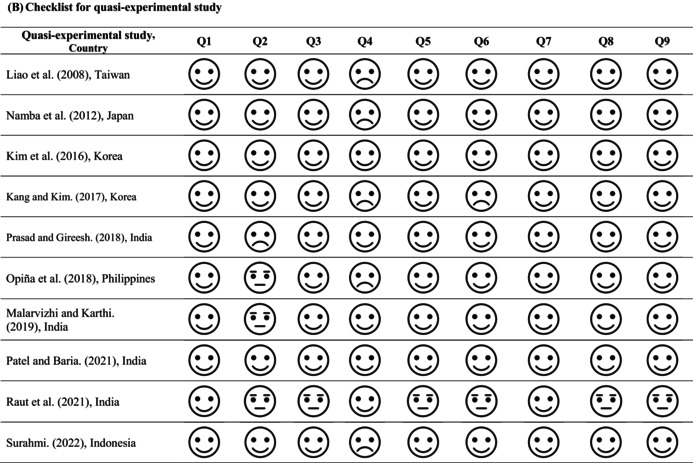

*Note:* 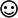 = criterion met (‘Yes’); 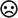 = criterion not met (‘No’); 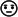 = insufficient information; (‘Unclear’). Bias domains: Selection bias (Q 1, 2, 4); Performance bias (Q 3); Detection bias (Q 5, 7, 8); Attrition bias (Q 6); Statistical conclusion validity (Q 9).

### 3.7 Effects of Footbaths on the Subjective Sleep Quality of Older Adults: Questionnaires

**FIGURE 2:**
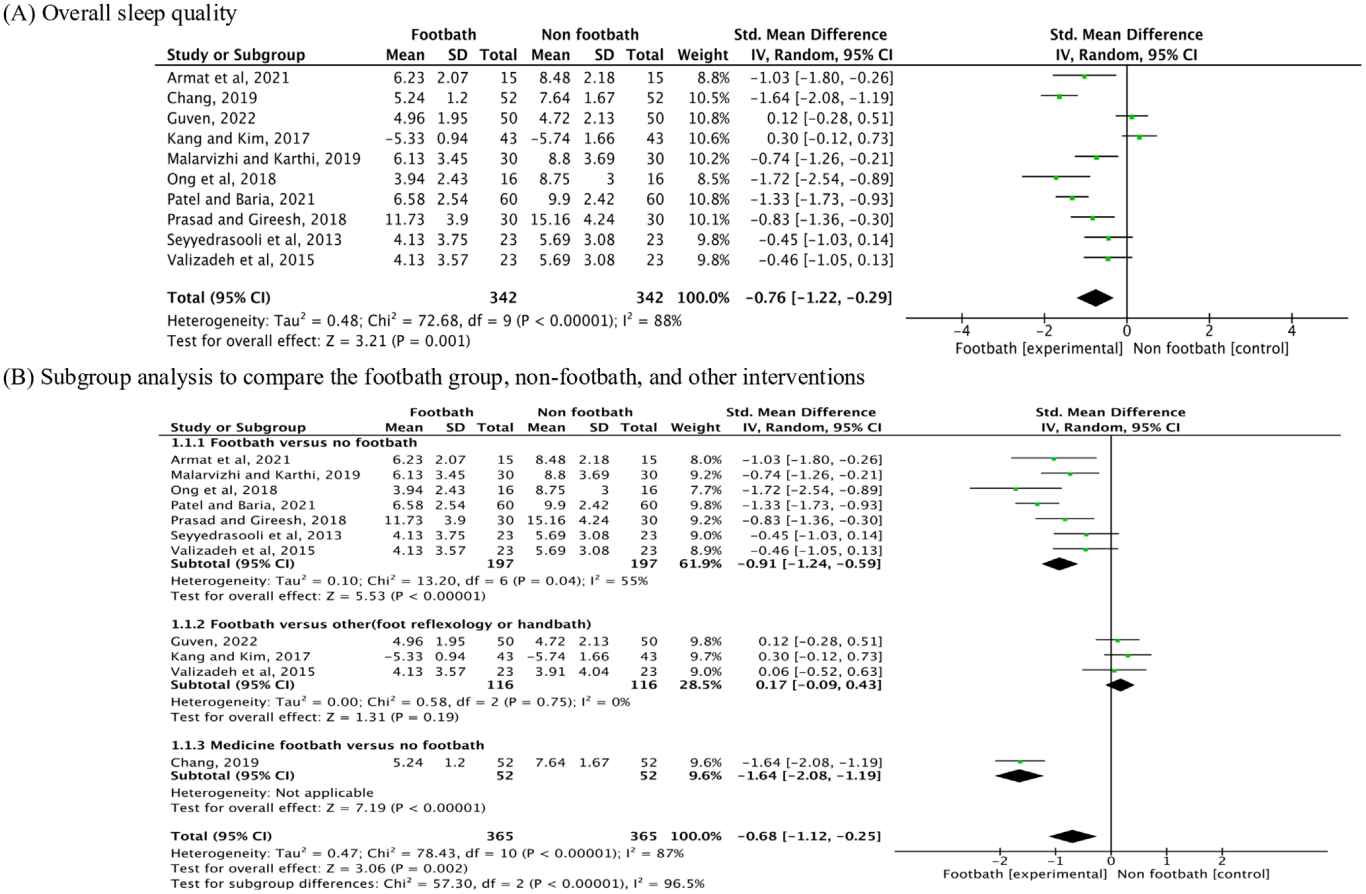
Standardised mean difference in sleep quality between footbaths and controls (non‐footbath or reflexology, hand‐bath) in older. (A) Overall sleep quality and (B) subgroup analysis by comparator condition.

Of the 18 included studies, ten (663 participants) were eligible for the meta‐analysis of subjective sleep quality. Figure 2A shows the comparison between the footbath and no footbath groups, which included other control interventions such as reflexology, hand baths and medication. The pooled results demonstrated that footbaths significantly improved the sleep quality of older adults compared with no footbath or other control interventions (SMD = −0.76, 95% CI: [−1.22, −0.29], *p* = 0.001). However, high heterogeneity (*I* ^2^ = 88%) was detected.

Owing to the high heterogeneity observed, subgroup analysis was conducted to explore the nuances of the intervention and control comparator conditions, as presented in Figure 2B. Among the ten studies, seven (416 participants) demonstrated a significant improvement in sleep quality among older adults receiving footbaths compared with those in the non‐footbath (standard care) group (SMD = −0.91, 95% CI: [−1.24, −0.59], *p* < 0.001). Notably, the heterogeneity was reduced from 87% to 55% after subgrouping. Additionally, one study revealed that footbaths incorporating traditional Chinese medicine significantly improved sleep quality compared with the non‐footbath group (SMD = −1.64, 95% CI: [−2.08, −0.25], *p* < 0.001). However, no significant differences were observed between footbaths and other active interventions such as foot reflexology or hand baths (SMD = 0.17, 95% CI: [−0.09, 0.43], *p* = 0.19).

### 3.8 Effects of Footbaths on the Objective Sleep Quality of Older Adults: PSG and ATG

**FIGURE 3:**
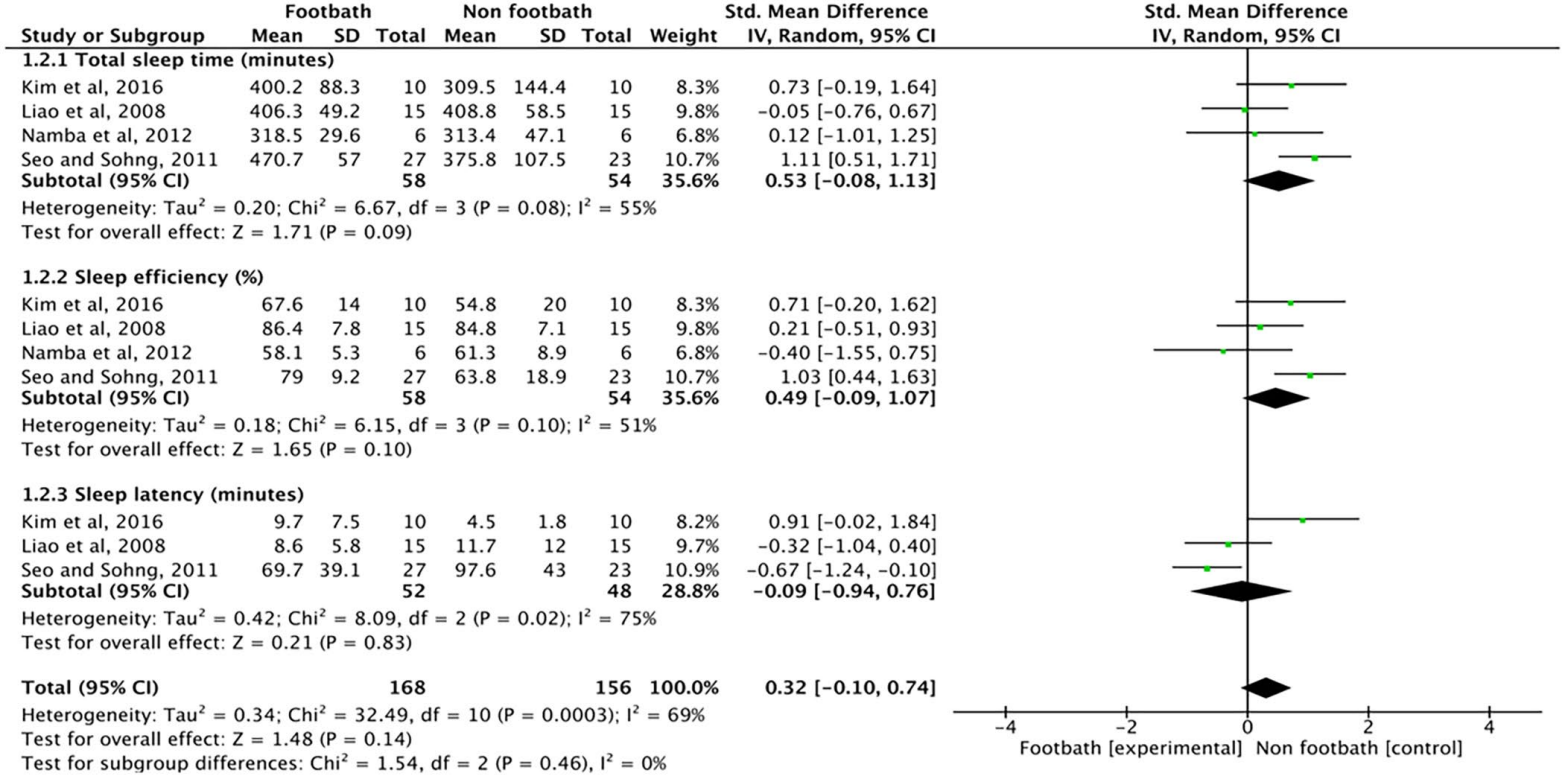
Standardised mean difference in the effect of footbaths on objective sleep quality among older adults: polysomnography and actigraphy.

As shown in Figure 3, objective sleep parameters assessed by PSG and ATG were further analysed. The pooled results indicated that footbaths, compared to non‐footbath controls, had no significant effects on total sleep time, sleep efficiency or sleep latency.

### 3.9 The Optimal Use of Footbaths

**FIGURE 4:**
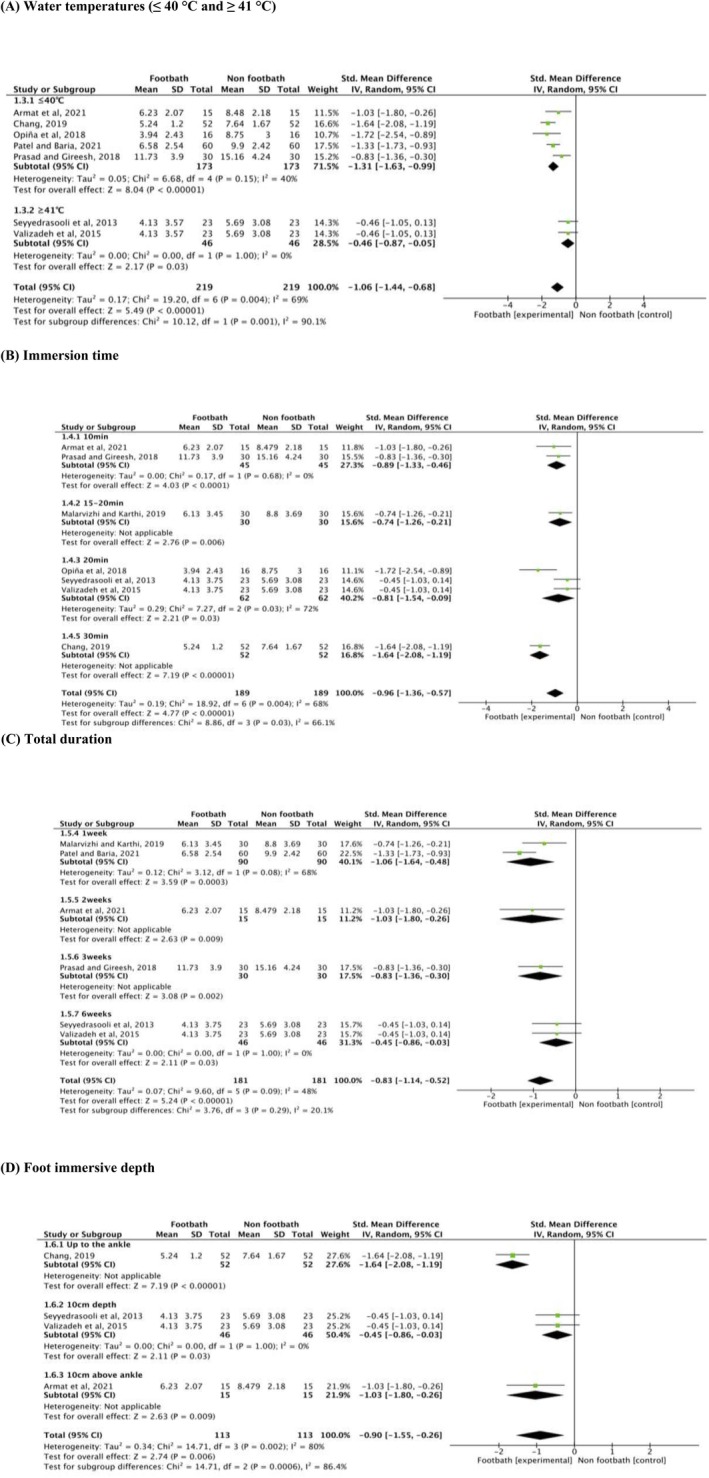
Subgroup analysis for optimal use of footbaths among older adults. (A) Water temperatures (≤ 40°C and ≥ 41°C); (B) immersion time; (C) total duration; and (D) foot immersive depth.

Figure 4A shows that older adults receiving footbaths at temperatures ≤ 40°C experienced significantly greater improvements in sleep quality compared to those in the non‐footbath group (SMD = −1.31, 95% CI: [−1.63, −0.99], *p* < 0.001).

Figure 4B illustrates significant effects of footbaths across immersion times ranging from 10 to 30 min (SMD = −0.96, 95% CI: [−1.36, −0.57], *p* < 0.001). The pooled meta‐analysis suggests that an immersion duration of at least 10 min is effective in improving sleep quality among older adults.

Figure 4C shows a significant effect of footbaths after only one week (SMD = −1.06, 95% CI: [−1.64, −0.48], *p* < 0.001). However, the effect diminished as the intervention duration increased. Nevertheless, even with a duration of six weeks, a significant effect was still observed (SMD = −0.45, 95% CI: [−0.86, −0.03], *p* = 0.03).

Figure 4D shows that a significant effect was observed when the immersion depth reached or exceeded the ankles (SMD = −1.64, 95% CI: [−2.08, −1.19], *p* < 0.001).

### 3.10 Reporting Bias Assessment

As shown in Figure S1, the funnel plot data is primarily concentrated on the left side of the horizontal axis and exhibits an asymmetric distribution, suggesting potential publication bias.

### 3.11 GRADE Assessment and Summary of Findings

The GRADE assessment (Table S3) showed that the overall CoE for the effectiveness of footbaths on sleep outcomes ranged from low to very low. The primary reasons for downgrading were a high risk of bias across most studies due to unclear randomization, lack of blinding and insufficient outcome assessment, as well as imprecision from small sample sizes and wide confidence intervals. For risk of bias, sleep quality and sleep latency were rated very serious, whereas sleep efficiency showed no serious concerns. Inconsistency was notable for sleep quality (*I* ^2^ > 50%), while other outcomes demonstrated acceptable homogeneity. All outcomes were rated not serious for indirectness, suggesting good alignment with the review objectives. Serious imprecision was identified for all outcomes, and publication bias was strongly suspected. Overall, the certainty of evidence (CoE) for the effectiveness of footbaths in improving sleep among older adults was rated as very low, mainly due to the high risk of bias, heterogeneity and limited sample sizes. The CoE for individual outcomes, such as sleep time, efficiency and latency, ranged from low to very low, reflecting uncertainty caused by methodological limitations and imprecision.

## 4 Discussion

The current review demonstrated that warm footbaths improved sleep quality in older adults. These findings align with previous systematic reviews on warm footbaths [13, 17], which reported significant improvements in sleep quality and quality of life among older adults. Furthermore, greater effectiveness was observed when water temperatures were maintained at ≤ 40°C (ranging from 35°C to 40°C), immersion depth reached the ankles, sessions lasted at least 10 min, and the intervention period was one week. These optimal conditions are consistent with those reported by Jiang, Chen and Belcastro [17], underscoring their potential importance in maximising intervention outcomes.

The beneficial effects of footbaths on sleep quality have often been attributed to physiological mechanisms. For example, peripheral vasodilation has been shown to enhance heat dissipation and promote sleep onset by increasing the distal–proximal skin temperature gradient (DPG) [42, 43, 44]. However, in this review, significant improvements were identified only in subjective sleep quality, while objective measures such as ATG and PSG revealed no significant changes. This discrepancy underscores the challenges of evaluating sleep outcomes, as objective instruments may not fully capture the psychological and emotional dimensions of restfulness and perceived well‐being [45]. Kolcaba's comfort theory provides a valuable framework for interpreting these findings. It conceptualises comfort as a holistic experience that includes physical, psychospiritual, environmental and sociocultural dimensions [14]. From this perspective, the observed improvements in sleep quality may reflect the psychospiritual benefits of footbaths, such as relaxation, reduced anxiety, emotional security, and their role as a ritualised self‐care practice, rather than measurable changes in sleep architecture or duration [33, 46]. The included studies provide information on the complexity of assessing sleep quality and the likelihood of discrepancies existing between subjective and objective evaluations. They highlight the importance of incorporating both measurement types in sleep research and emphasise the necessity for additional studies to more thoroughly comprehend these variances.

Notably, most studies included in this review were conducted in Asian countries, particularly China, Iran and India, where footbaths are deeply rooted in cultural traditions and widely regarded as complementary and alternative medicine. This cultural familiarity may further amplify the intervention's effects, as individuals are often more receptive and adherent to practices embedded in their cultural context [47]. Engaging in culturally meaningful activities has also been linked to enhanced emotional well‐being and stronger intervention outcomes [48]. These influences align with the sociocultural dimension of Kolcaba's comfort theory, which emphasises that comfort is shaped not only by physical and psychological factors but also by cultural context [14].

Although the cultural alignment of footbaths may limit generalisability, it is noteworthy that Asian medical traditions have gained increasing recognition and integration within Western healthcare systems. You, Zhang and Liu [49] reported that traditional Chinese medicine has been widely adopted in the United States, the United Kingdom and Europe as adjunctive therapy for disease management, immune support and symptom relief. To further expand the applicability of culturally rooted interventions such as footbaths, future research should explore their acceptability and effectiveness in diverse geographic and cultural contexts. Concurrently, developing standardised clinical guidelines and protocols will be crucial for facilitating their integration into holistic and patient‐centred care models.

Finally, this review found no significant differences between the effects of footbaths, foot reflexology and hand baths on sleep quality among older adults. This similarity may be explained by shared mechanisms such as thermal comfort, relaxation and autonomic regulation [50]. However, we found that footbaths combined with traditional Chinese medicine significantly enhanced sleep quality in older adults compared with warm water footbaths alone. A possible explanation is that these interventions may act through comparable mechanisms involving thermal comfort, relaxation and autonomic regulation [51]. Additionally, this improvement may result from synergistic effects, as certain Chinese medicinal herbs contain natural compounds with sedative and calming properties that act on the nervous system [52]. Further high‐quality RCTs are needed to isolate and quantify the individual and combined effects of thermal stimulation and herbal components in sleep improvement interventions.

### 4.1 Strengths and Limitations

Our study was the first to comprehensively combine subjective and objective sleep quality parameters to evaluate the effects and optimal procedures of footbaths on the sleep quality of older adults. However, several limitations of this study need to be acknowledged. First, the sample sizes of the included studies were relatively small, and some lacked detailed information on footbath parameters, such as water temperature, immersion depth and immersion duration, which limited a comprehensive evaluation of the intervention's effects. In addition, most studies have been conducted in Asian cultural contexts, which may limit the generalisability of the findings to populations in other regions with different cultural practices and attitudes towards self‐care interventions. Second, the nature of footbaths and the use of subjective assessments in the included studies, which could inevitably lead to placebo effects in older adults, represented a potential additional limitation. Finally, this study may be susceptible to reporting bias, even though we have thoroughly explored the evidence. Certain nonsignificant outcomes were not fully detailed in the results section.

## 5 Conclusion

Warm water footbaths prior to bedtime are a popular practice recognised for improving sleep quality. On the basis of the evidence from this review, we suggest that standardised footbath protocols could be developed using warm water at temperatures ≤ 40°C, with an immersion duration of at least 10 min, ideally administered approximately one hour before bedtime daily for a minimum of one week. These parameters could be integrated into institutional guidelines for sleep hygiene and nonpharmacological interventions in geriatric care settings. Future research should prioritise large‐scale, multicentre trials designed to incorporate both subjective and objective measurements for a more comprehensive evaluation of sleep outcomes. Additionally, cross‐cultural comparative studies are needed to examine how cultural context influences the acceptability and perceived effectiveness of such interventions. Finally, there is a need to develop theoretical models, such as those grounded in comfort theory or self‐care frameworks, to better explain the observed discrepancies between subjective improvements and objective sleep measures and to guide the design of future interventions.

## Author Contributions

S.‐Y. Chang, Y.‐C. Lee, Y.‐C. Chen, C.‐T. Chen, C.‐J. Fang and H.‐Y. Hung were responsible for the study design. S.‐Y. Chang, Y.‐C. Lee, Y.‐C. Chen, C.‐T. Chen and H.‐Y. Hung completed the data analysis and manuscript preparation. S.‐Y. Chang, Y.‐C. Lee, Y.‐C. Chen, C.‐T. Chen and H.‐Y. Hung approved the final manuscript.

## Disclosure

The authors have nothing to report.

## Ethics Statement

This study was exempted from full ethical review by the Research Ethics Committee of Cheng Kung University and Hospital International Review Board (IRB approval number: B‐ER‐111‐214).

## Conflicts of Interest

The authors declare no conflicts of interest.

## Supporting information


**Table S1:** PRISMA 2020 checklist.
**Table S2:** Search strategy.
**Table S3:** Grading of Recommendations, Assessment, Development, and Evaluation (GRADE) assessment and summary of findings.

**Figure S1:** Funnel plot for assessing publication bias.

## Data Availability

The data that support the findings of this study are available from the corresponding authors upon reasonable request.
