# Supplementary material for: A Systematic Review and Meta‐Analysis of Footbath Effects and Optimal Procedures to Improve Sleep in Older Adults
Source: Scand J Caring Sci. 2025 Sep 19;39(3):e70118. doi: 10.1111/scs.70118 (PMC12449616; doi:10.1111/scs.70118)
Supplement: Supplementary file 1 — Table S1: PRISMA 2020 checklist. Table S2: Search strategy. Table S3: Grading of Recommendations, Assessment, Development, and Evaluation (GRADE) assessment and summary of findings. [file SCS-39-0-s001.docx]

**SUPPLEMENTARY**

**Table S1.** PRISMA 2020 Checklist

| **Section and Topic** | **Item #** | **Checklist item** | **Location where item is reported** |
| --- | --- | --- | --- |
| **TITLE** | | |  |
| Title | 1 | Identify the report as a systematic review. | Page 1 |
| **ABSTRACT** | | |  |
| Abstract | 2 | See the PRISMA 2020 for Abstracts checklist. | Page 1-2 |
| **INTRODUCTION** | | |  |
| Rationale | 3 | Describe the rationale for the review in the context of existing knowledge. | Page 3-4 |
| Objectives | 4 | Provide an explicit statement of the objective(s) or question(s) the review addresses. | Page 4 |
| **METHODS** | | |  |
| Eligibility criteria | 5 | Specify the inclusion and exclusion criteria for the review and how studies were grouped for the syntheses. | Page 5-6 |
| Information sources | 6 | Specify all databases, registers, websites, organisations, reference lists and other sources searched or consulted to identify studies. Specify the date when each source was last searched or consulted. | Page 4 |
| Search strategy | 7 | Present the full search strategies for all databases, registers and websites, including any filters and limits used. | Page 4 and  supplementary table 2 |
| Selection process | 8 | Specify the methods used to decide whether a study met the inclusion criteria of the review, including how many reviewers screened each record and each report retrieved, whether they worked independently, and if applicable, details of automation tools used in the process. | Page 5-6 |
| Data collection process | 9 | Specify the methods used to collect data from reports, including how many reviewers collected data from each report, whether they worked independently, any processes for obtaining or confirming data from study investigators, and if applicable, details of automation tools used in the process. | Page 5-6 |
| Data items | 10a | List and define all outcomes for which data were sought. Specify whether all results that were compatible with each outcome domain in each study were sought (e.g. for all measures, time points, analyses), and if not, the methods used to decide which results to collect. | Page 6 |
|  | 10b | List and define all other variables for which data were sought (e.g. participant and intervention characteristics, funding sources). Describe any assumptions made about any missing or unclear information. | Page 6 and table 1 |
| Study risk of bias assessment | 11 | Specify the methods used to assess risk of bias in the included studies, including details of the tool(s) used, how many reviewers assessed each study and whether they worked independently, and if applicable, details of automation tools used in the process. | Page 6 and  table 2 |
| Effect measures | 12 | Specify for each outcome the effect measure(s) (e.g. risk ratio, mean difference) used in the synthesis or presentation of results. | Page 7 |
| Synthesis methods | 13a | Describe the processes used to decide which studies were eligible for each synthesis (e.g. tabulating the study intervention characteristics and comparing against the planned groups for each synthesis (item #5)). | Page 7-8 |
|  | 13b | Describe any methods required to prepare the data for presentation or synthesis, such as handling of missing summary statistics, or data conversions. | NA |
|  | 13c | Describe any methods used to tabulate or visually display results of individual studies and syntheses. | NA |
|  | 13d | Describe any methods used to synthesize results and provide a rationale for the choice(s). If meta-analysis was performed, describe the model(s), method(s) to identify the presence and extent of statistical heterogeneity, and software package(s) used. | Page 7-8 |
|  | 13e | Describe any methods used to explore possible causes of heterogeneity among study results (e.g. subgroup analysis, meta-regression). | Page 7-8 |
|  | 13f | Describe any sensitivity analyses conducted to assess robustness of the synthesized results. | NA |
| Reporting bias assessment | 14 | Describe any methods used to assess risk of bias due to missing results in a synthesis (arising from reporting biases). | Page 6 and Supplementary Figure 1 |
| Certainty assessment | 15 | Describe any methods used to assess certainty (or confidence) in the body of evidence for an outcome. | Page 7 and Supplementary Table 3 |
| **RESULTS** | | |  |
| Study selection | 16a | Describe the results of the search and selection process, from the number of records identified in the search to the number of studies included in the review, ideally using a flow diagram. | Page 7 and Figure 1 |
|  | 16b | Cite studies that might appear to meet the inclusion criteria, but which were excluded, and explain why they were excluded. | Page 7 |
| Study characteristics | 17 | Cite each included study and present its characteristics. | Page 7 and Table 1 |
| Risk of bias in studies | 18 | Present assessments of risk of bias for each included study. | Page 12-13, Table 2, and Supplementary table 3 |
| Results of individual studies | 19 | For all outcomes, present, for each study: (a) summary statistics for each group (where appropriate) and (b) an effect estimate and its precision (e.g. confidence/credible interval), ideally using structured tables or plots. | Table 1 |
| Results of syntheses | 20a | For each synthesis, briefly summarise the characteristics and risk of bias among contributing studies. | Page 10-12, Table 2, and Supplementary table 3 |
|  | 20b | Present results of all statistical syntheses conducted. If meta-analysis was done, present for each the summary estimate and its precision (e.g. confidence/credible interval) and measures of statistical heterogeneity. If comparing groups, describe the direction of the effect. | Figure 2-4 |
|  | 20c | Present results of all investigations of possible causes of heterogeneity among study results. | Page 11 |
|  | 20d | Present results of all sensitivity analyses conducted to assess the robustness of the synthesized results. | NA |
| Reporting biases | 21 | Present assessments of risk of bias due to missing results (arising from reporting biases) for each synthesis assessed. | Page 12 |
| Certainty of evidence | 22 | Present assessments of certainty (or confidence) in the body of evidence for each outcome assessed. | Page 12-13 |
| **DISCUSSION** | | |  |
| Discussion | 23a | Provide a general interpretation of the results in the context of other evidence. | Page 13-15 |
|  | 23b | Discuss any limitations of the evidence included in the review. | Page 15 |
|  | 23c | Discuss any limitations of the review processes used. | NA |
|  | 23d | Discuss implications of the results for practice, policy, and future research. | Page 16 |
| **OTHER INFORMATION** | | |  |
| Registration and protocol | 24a | Provide registration information for the review, including register name and registration number, or state that the review was not registered. | Abstract and Page 4 |
|  | 24b | Indicate where the review protocol can be accessed, or state that a protocol was not prepared. | Page 4 |
|  | 24c | Describe and explain any amendments to information provided at registration or in the protocol. | NA |
| Support | 25 | Describe sources of financial or non-financial support for the review, and the role of the funders or sponsors in the review. | Page 17 |
| Competing interests | 26 | Declare any competing interests of review authors. | Page 17 |
| Availability of data, code and other materials | 27 | Report which of the following are publicly available and where they can be found: template data collection forms; data extracted from included studies; data used for all analyses; analytic code; any other materials used in the review. | Page 17 |

**Table S2.** Search strategy

| **Database** | **#** | **Search syntax** | **Number of articles** |
| --- | --- | --- | --- |
| 1) Embase | 1 | (elder* OR aged OR aging OR geriatric* OR gerontolog* OR senior* OR senium* OR "old age*" OR (older NEAR/2 (adult* OR person* OR people OR patient* OR population* OR men OR women OR male OR female OR subject* OR citizen*))):ti,ab,kw | 1,970,857 |
|  | 2 | "aging"/exp OR "aged"/exp OR "geriatrics"/exp OR "gerontology"/exp OR "elderly care"/exp | 4,092,033 |
|  | 3 | (footbath* OR bath* OR "hot spring*" OR "geothermal spring*" OR hydrotherap* OR spa OR spas OR balneotherap* OR "balneo therap*" OR balneolog* OR sauna* OR warm* OR heat* OR hyperthermia OR thermotherap* OR "high temperature*" OR "hot temperature*"):ti,ab,kw | 652,397 |
|  | 4 | "bath"/exp OR "thermal spring"/exp OR "balneotherapy"/exp OR "heat"/exp OR "heating"/exp OR "hyperthermia"/exp OR "thermotherapy"/exp OR "high temperature"/exp OR "warming"/exp | 276,742 |
|  | 5 | (sleep* OR insomni* OR dyssomni* OR agrypni* OR hyposomni* OR sopor* OR parasomni* OR polysomnograph*):ti,ab,kw | 387,336 |
|  | 6 | "sleep disorder"/exp OR "sleep study"/exp OR "sleep"/exp OR "sleep parameters"/exp OR "sleep hygiene"/exp OR "sleep deprivation"/exp | 503,493 |
|  | 7 | (#1 OR #2) AND (#3 OR #4) AND (#5 OR #6) AND [embase]/lim | 1,200 |
|  | 8 | #7 AND ("randomized controlled trial"/de or "controlled clinical trial"/de or "randomization"/de or "intermethod comparison"/de or "double blind procedure"/de or "human experiment"/de OR (random* or placebo or assigned or allocated or volunteer or volunteers or (open NEXT/1 label) or ((double or single or doubly or singly) NEXT/1 (blind or blinded or blindly)) or "parallel group?" or crossover or "cross over" or ((assign* or match or matched or allocation) NEAR/5 (alternate or group? or intervention? or patient? or subject? or participant?)) OR (controlled NEAR/7 (study or design or trial))):ti,ab OR (compare or compared or comparison or trial):ti OR ((evaluated or evaluate or evaluating or assessed or assess) and (compare or compared or comparing or comparison)):ab) NOT (((random* NEXT/1 sampl* NEAR/7 ("cross section*" or questionnaire? or survey* or database?)):ti,ab not ("comparative study"/de or "controlled study"/de or "randomi?ed controlled":ti,ab or "randomly assigned":ti,ab)) OR ("Cross-sectional study"/de not ("randomized controlled trial"/de or "controlled clinical study"/de or "controlled study"/de or randomi?ed controlled:ti,ab or "control group?":ti,ab)) OR ((((case NEXT/1 control*) and random*) not randomi?ed controlled):ti,ab) OR ("Systematic review" not (trial or study)):ti OR (nonrandom* not random*):ti,ab OR "Random field*":ti,ab OR ("random cluster" NEAR/3 sampl*):ti,ab OR ((review:ab and review/it) not trial:ti) OR ("we searched":ab and (review:ti or review/it)) OR "update review":ab OR (databases NEAR/4 searched):ab OR ((rat or rats or mouse or mice or swine or porcine or murine or sheep or lambs or pigs or piglets or rabbit or rabbits or cat or cats or dog or dogs or cattle or bovine or monkey or monkeys or trout or marmoset?):ti and "animal experiment"/de) OR ("animal experiment"/de not ("human experiment"/de or "human"/de)))  Filter Source: Box 3.e., [Technical Supplement to Chapter 4: Searching for and Selecting Studies.](https://training.cochrane.org/handbook/version-6/chapter-4-tech-suppl) Cochrane Handbook for Systematic Reviews of Interventions Version 6. (Syntax Translated from Ovid Embase to Elsevier Embase.com.) | 392 |
| 2) Medline ovid | 1 | (elder* OR aged OR aging OR geriatric* OR gerontolog* OR senior* OR senium* OR "old age*" OR (older ADJ2 (adult* OR person* OR people OR patient* OR population* OR men OR women OR male OR female OR subject* OR citizen*))).mp | 6,395,351 |
|  | 2 | exp "aging"/ OR exp "aged"/ OR exp "geriatrics"/ OR exp "geriatric nursing" | 3,684,940 |
|  | 3 | (footbath* OR bath* OR "hot spring*" OR "geothermal spring" OR hydrotherap* OR balneotherap* OR "balneo therap*" OR spa OR spas OR balneolog* OR sauna* OR warm* OR heat* OR hyperthermia OR thermotherap* OR "hot temperature*" OR "high temperature*").mp | 673,530 |
|  | 4 | exp "baths"/ OR exp "balneology"/ OR exp "hot springs"/ OR exp "hydrotherapy"/ OR exp "hyperthermia"/ OR exp "hot temperature"/ | 164,670 |
|  | 5 | (sleep* OR dyssomni* OR sopor* OR insomni* OR agrypni* OR hyposomni* OR parasomni* OR polysomnograph*).mp | 277,817 |
|  | 6 | exp "dyssomnias"/ OR exp "sleep"/ OR exp "sleep quailty"/ OR exp "sleep hygiene"/ OR exp "sleep wake disorders"/ OR exp "sleep latency"/ OR exp "sleep deprivation"/ OR exp "sleep initiation and maintenance disorders"/ OR exp "sleep stages"/ OR exp "polysomnography"/ OR exp "parasomnias"/ OR exp "sleep, REM"/ | 175,694 |
|  | 7 | (1 OR 2) AND (3 OR 4) AND (5 OR 6) | 911 |
|  | 8 | 7 AND (randomized controlled trial.pt or controlled clinical trial.pt or randomi*ed.ab or placebo.ab. or drug therapy.fs. or randomly.ab. or trial.ab. or groups.ab. not (exp animals/ not humans.sh.)) | 360 |
| 3) Cochrane | 1 | (elder* OR aged OR aging OR geriatric* OR gerontolog* OR senior* OR senium* OR (old NEXT age*) OR (older NEAR/2 (adult* OR person* OR people OR patient* OR population* OR men OR women OR male OR female OR subject* OR citizen*))):ti,ab,kw | 657,673 |
|  | 2 | [mh "aging"] OR [mh "aged"] OR [mh "geriatric nursing"] OR [mh "geriatrics"] | 257,780 |
|  | 3 | (footbath* OR bath* OR (hot NEXT spring*) OR (geothermal NEXT spring) OR hydrotherap* OR spa OR spas OR (balneo NEXT therap*) OR balneolog* OR balneotherap* OR sauna* OR warm* OR heat* OR hyperthermia OR thermotherap* OR (hot NEXT temperature*) OR (high NEXT temperature*)):ti,ab,kw | 32,952 |
|  | 4 | [mh "baths"] OR [mh "balneology"] OR [mh "hot springs"] OR [mh "hydrotherapy"] OR [mh "hyperthermia"] OR [mh "hot temperature"] | 4,752 |
|  | 5 | (sleep* OR dyssomn* OR sopor* OR insomni* OR agrypni* OR hyposomni* OR parasomni* OR polysomnograph*):ti,ab,kw | 60,888 |
|  | 6 | [mh "dyssomnias"] OR [mh "sleep"] OR [mh "sleep quailty"] OR [mh "sleep hygiene"] OR [mh "sleep wake disorders"] OR [mh "sleep latency"] OR [mh "sleep deprivation"] OR [mh "sleep initiation and maintenance disorders"] OR [mh "sleep stages"] OR [mh "polysomnography"] OR [mh "sleep, REM"] OR [mh "parasomnias"] | 17,585 |
|  | 7 | (#1 OR #2) AND (#3 OR #4) AND (#5 OR #6) | - |
|  | 8 | #7 Limits: in Trials | 513 |
| 4) CINAHL EBSCOhost | 1 | elder* OR aged OR aging OR geriatric* OR gerontolog* OR senior* OR senium* OR "old age*" OR (older N1 (adult* OR person* OR people OR patient* OR population* OR men OR women OR male OR female OR subject* OR citizen*)) | 4,983,978 |
|  | 2 | mh ("aged+" OR "geriatrics+" OR "geriatric nursing+" OR "aging+ OR "gerontologic care+") | 962,201 |
|  | 3 | footbath*OR bath* OR "hot spring*" OR "geothermal spring" OR hydrotherap* OR spa OR spas OR "balneo therap*" OR balneolog* OR balneotherap* OR sauna* OR warm* OR heat* OR hyperthermia OR thermotherap* OR "hot temperature*" OR "high temperature*" | 86,100 |
|  | 4 | mh ("bathing and baths+" OR "hydrotherapy+" OR "balneology+" OR "warming techniques+" OR "heat+" OR "hyperthermia+" OR "skin temperature+") | 22,910 |
|  | 5 | sleep* OR dyssomn* OR sopor* OR insomni* OR agrypni* OR hyposomni* OR parasomni* OR polysomnograph* | 103,659 |
|  | 6 | mh ("sleep disorders+" OR "sleep+" OR "sleepiness+" OR "polysomnography+") | 69,158 |
|  | 7 | (#1 OR #2) AND (#3 OR #4) AND (#5 OR #6) | 44 |
| 5) Scopus | 1 | title-abs (elder* OR aged OR aging OR geriatric* OR gerontolog* OR senior* OR senium* OR "old age*" OR (older w/1 (adult* OR person* OR people OR patient* OR population* OR men OR women OR male OR female OR subject* OR citizen*))) OR authkey (elder* OR aged OR aging OR geriatric* OR gerontolog* OR senior* OR senium* OR "old age*" OR (older w/1 (adult* OR person* OR people OR patient* OR population* OR men OR women OR male OR female OR subject* OR citizen*))) | 2,207,631 |
|  | 2 | title-abs (footbath* or bath* or "hot spring*" or "geothermal spring" or hydrotherap* or spa or spas or "balneo therap*" or balneolog* or balneotherap* or sauna* or warm* or heat* or hyperthermia or thermotherap* or "hot temperature*" or "high temperature*") OR authkey (footbath* or bath* or "hot spring*" or "geothermal spring" or hydrotherap* or spa or spas or "balneo therap*" or balneolog* or balneotherap* or sauna* or warm* or heat* or hyperthermia or thermotherap* or "hot temperature*" or "high temperature*") | 3,380,088 |
|  | 3 | title-abs (sleep* OR dyssomn* OR sopor* OR insomni* OR agrypni* OR hyposomni* OR parasomni* OR polysomnograph*) OR authkey (sleep* OR dyssomn* OR sopor* OR insomni* OR agrypni* OR hyposomni* OR parasomni* OR polysomnograph*) | 348,045 |
|  | 4 | (INDEXTERMS ( "clinical trials" OR "clinical trials as a topic" OR "randomized controlled trial" OR "Randomized Controlled Trials as Topic" OR "controlled clinical trial" OR "Controlled Clinical Trials" OR "random allocation" OR "Double-Blind Method" OR "Single-Blind Method" OR "Cross-Over Studies" OR "Placebos" OR "multicenter study" OR "double blind procedure" OR "single blind procedure" OR "crossover procedure" OR "clinical trial" OR "controlled study" OR "randomization" OR "placebo" ) OR TITLE-ABS-KEY ( "clinical trials" OR "clinical trials as a topic" OR "randomized controlled trial" OR "Randomized Controlled Trials as Topic" OR "controlled clinical trial" OR "Controlled Clinical Trials as Topic" OR "random allocation" OR "randomly allocated" OR "allocated randomly" OR "Double-Blind Method" OR "Single-Blind Method" OR "Cross-Over Studies" OR "Placebos" OR "cross-over trial" OR "single blind" OR "double blind" OR "factorial design" OR "factorial trial" ) OR TITLE ( clinical trial OR trial OR rct* OR random* OR blind* )) | 4,752 |
|  | 5 | #1 AND #2 AND #3 AND #4 | 142 |
| 6) Web of science | 1 | TS=(elder* OR aged OR aging OR geriatric* OR gerontolog* OR senior* OR senium* OR "old age*" OR (older NEAR/1 (adult* OR person* OR people OR patient* OR population* OR men OR women OR male OR female OR subject* OR citizen*))) | 4,752,125 |
|  | 2 | TS=(footbath* OR bath* OR "hot spring*" OR "geothermal spring" OR hydrotherap* OR spa OR spas OR "balneo therap*" OR balneolog* OR balneotherap* OR sauna* OR warm* OR heat* OR hyperthermia OR thermotherap* OR "hot temperature*" OR "high temperature*") | 2,322,539 |
|  | 3 | TS=(sleep* OR dyssomn* OR sopor* OR insomni* OR agrypni* OR hyposomni* OR parasomni* OR sopor OR polysomnograph*) | 338,449 |
|  | 4 | #1 AND #2 AND #3 | 985 |
|  | 5 | #4 AND (TS= clinical trial* OR TS=research design OR TS=comparative stud* OR TS=evaluation stud* OR TS=controlled trial* OR TS=follow-up stud* OR TS=prospective stud* OR TS=random* OR TS=placebo* OR TS=(single blind*) OR TS=(double blind*)) | 304 |
| 7) Airiti library | 1 | 老人 OR 老年 OR 高齡 OR aged OR elder* OR aging OR geriatric* OR gerontolog* OR senior* OR senium* OR old age | 43,537 |
|  | 2 | 足浴 OR 泡腳 OR 溫水足浴 OR 溫泉 OR 浴療 OR footbath* OR bath* OR hot spring* OR hydrotherap* OR spa OR spas OR balneotherap* OR balneo therap* OR balneolog* OR sauna* OR warm* OR heat* OR hyperthermia OR thermotherap* OR high temperature* OR hot temperature* | 36,728 |
|  | 3 | 睡眠 OR 睡眠多項生理檢查 OR sleep* OR insomni* OR dyssomni* OR agrypni* OR hyposomni* OR sopor* OR parasomni* OR polysomnograph* | 4,988 |
|  | 4 | #1 AND #2 AND #3 | 10 |
| 8) China national knowledge infrastructure (CNKI) | 1 | SU = (老人 + 老年 + 高齡 + aged + elder + aging + geriatric + gerontolog + senior + senium + old age) | 940,896 |
|  | 2 | SU = (足浴 + 泡腳 + 溫水足浴 + 溫泉 + 浴療 + footbath + bath + "hot spring" + "geothermal spring" + hydrotherapy + spa + spas + balneotherapy + "balneo therapy" + balneology + sauna + warm + heat + hyperthermia + thermotherapy + "high temperature" + "hot temperature") | 621,516 |
|  | 3 | SU = (睡眠 + 睡眠多項生理檢查 + sleep + insomnia + dyssomnia + agrypnia + hyposomnia + sopor + parasomnia + polysomnography) | 162,465 |
|  | 4 | #1 AND #2 AND #3 | 72 |
|  | 5 | SU = (随机 + 对照 + 控制组 + 安慰剂 + 试验 + 分组 + 隨機 + 對照 + 控制組 + 安慰劑 + 試驗 + 分組 + random + randomly + randomized + randomized + placebo + trial + groups) | 16 |
| 9) Google scholar | 1 | (elder* OR aged OR aging OR geriatric* OR gerontolog* OR senior* OR senium* OR "old age*" OR older) AND (footbath* OR bath* OR "hot spring*" OR "geothermal spring" OR hydrotherap* OR spa OR spas OR "balneo therap*" OR balneolog* OR balneotherap* OR sauna* OR warm* OR heat* OR "local hyperthermia" OR thermotherap* OR "hot temperature*" OR "high temperature*") AND (sleep* OR dyssomn* OR sopor* OR insomni* OR agrypni* OR hyposomni* OR parasomni* OR sopor* OR polysomnograph*) AND (randomized OR RCT) | 1,010 |
|  | 2 | selection | 60 |

**Table S3**. Grading of Recommendations, Assessment, Development, and Evaluation (GRADE) assessment and summary of findings

| **Certainty assessment** | | | | | | | **Summary of findings** |
| --- | --- | --- | --- | --- | --- | --- | --- |
| **Participants (studies) Follow-up** | **Risk of bias** | **Inconsistency** | **Indirectness** | **Imprecision** | **Publication bias** | **Overall certainty of evidence** | **Risk difference with Footbath (95% CI)** |
| **Footbath compared to non-Footbath for sleep quality** | | | | | | | |
| 684 (10 studies) | Very  serious ^a^ | Very  serious ^b^ | Not  serious | Serious ^C^ | Publication bias strongly suspected | ⨁◯◯◯ Very low | SMD -0.76  (-1.22, -0.29) |
| **Footbath compared to non-Footbath for total sleep time** | | | | | | | |
| 112 (4 studies) | Very  serious ^a^ | Not  serious | Not  serious | Serious ^c^ | Publication bias strongly suspected | ⨁⨁◯◯  low | SMD 0.53  (-0.08, 1.13) |
| **Footbath compared to non-Footbath for sleep efficiency** | | | | | | | |
| 112 (4 studies) | Not  serious ^a^ | Not  serious | Not  serious | Serious ^C^ | Publication bias strongly suspected | ⨁⨁◯◯  low | SMD 0.49 (-0.09, 1.07) |
| **Footbath compared to non-Footbath for sleep latency** | | | | | | | |
| 100 (3 studies) | Very  serious ^a^ | Not  serious ^b^ | Not  serious | Serious ^c^ | Publication bias strongly suspected | ⨁◯◯◯ Very low | SMD - 0.09 (-0.94, 0.76) |

*Abbreviations:* CI =confidence interval; SMD= standardized mean difference; MD= mean difference; RCTs=randomized control trials.

Explanations

a. The majority of the included RCT/ quasi-RCTs had unclear information in concealment, blinding of participants or researcher, and outcome assessment. The risk of bias was downgraded two levels.

b. High heterogeneity.

c. Small simple size and wide range of 95% confidence interval.
