# Supplementary figures and images for: A Systematic Review and Meta‐Analysis of Footbath Effects and Optimal Procedures to Improve Sleep in Older Adults
Source: Scand J Caring Sci. 2025 Sep 19;39(3):e70118. doi: 10.1111/scs.70118 (PMC12449616; doi:10.1111/scs.70118)

**SUPPLEMENTARY**

**Figure S1.** Funnel plot for assessing publication bias


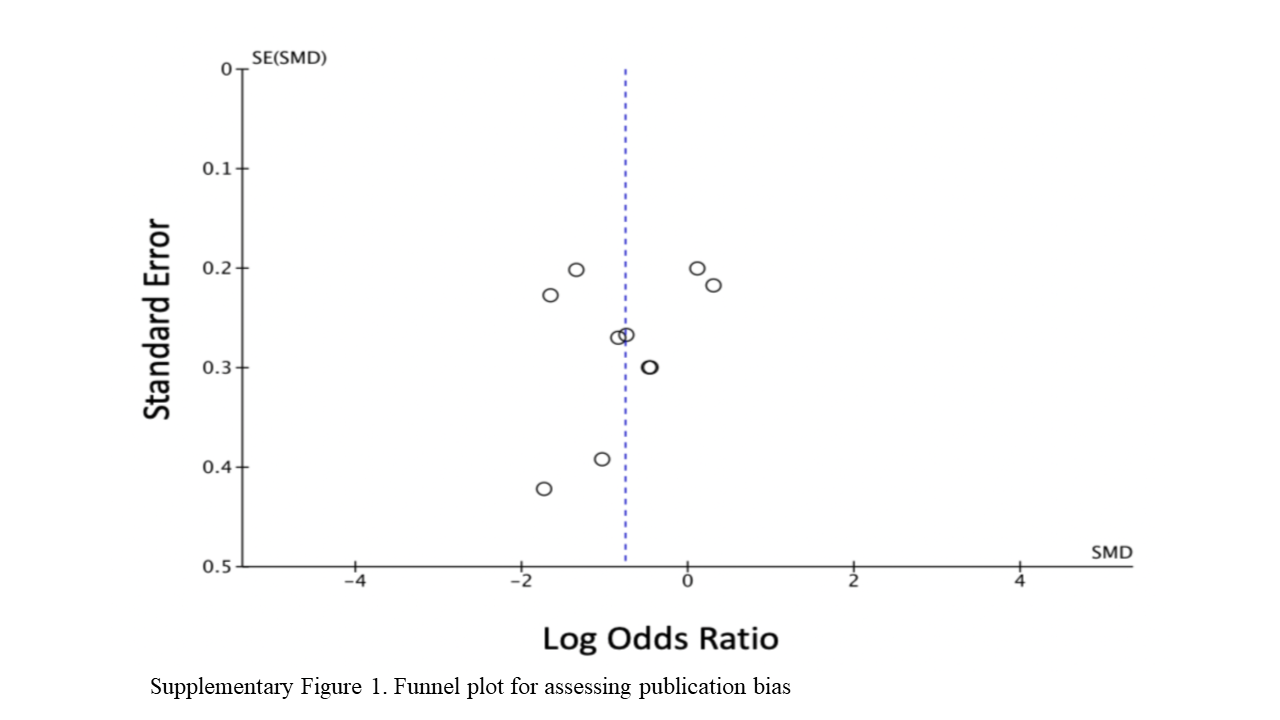

Supplement: Supplementary file 2 — Figure S1: Funnel plot for assessing publication bias. [file SCS-39-0-s002.docx]
